# Supplementary material for: A subject-specific reversible folding model reveals geometry-driven white-matter organization
Source: bioRxiv. 2025 Dec 12:2025.12.10.693407. Preprint. [Version 1] doi: 10.64898/2025.12.10.693407 (PMC12710974; doi:10.64898/2025.12.10.693407)
Supplement: 5 [file NIHPP2025.12.10.693407v1-supplement-5.pdf]

bioRxiv preprint doi: https://doi.org/10.64898/2025.12.10.693407; this version posted December 13, 2025. The copyright holder for this preprint (which was not certified by peer review) is the author/funder, who has granted bioRxiv a license to display the preprint in perpetuity. It is made available under aCC-BY 4.0 International license.

Supplementary Material

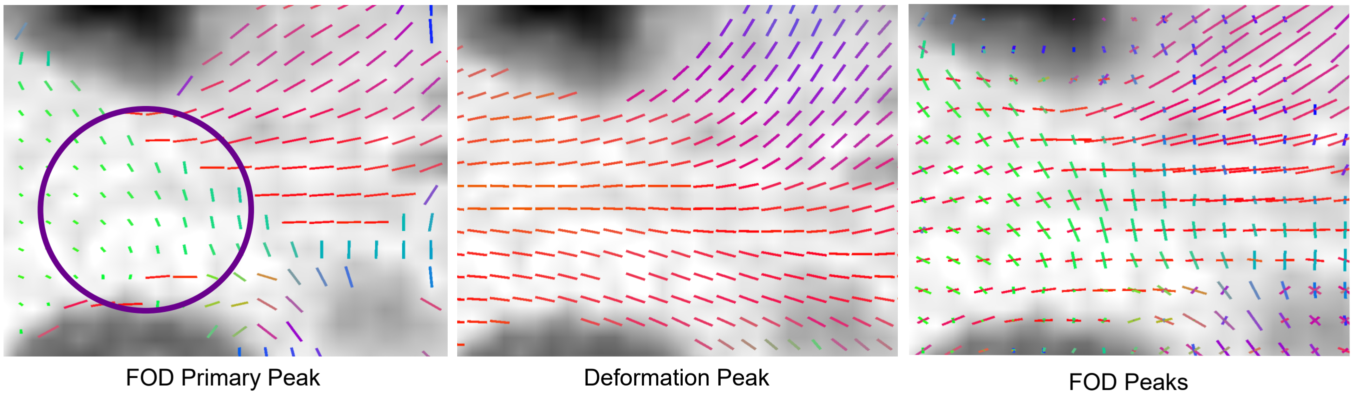

Figure 1: Region with low-scoring primary peak alignment (purple circle), where deformation-derived peaks align with secondary peaks.
